# Antibiotic-induced microbiota disruption impairs neutrophil-mediated immunity to respiratory *Aspergillus fumigatus* infection in mice

**DOI:** 10.1128/mbio.03982-25

**Published:** 2026-03-11

**Authors:** Mariano A. Aufiero, Tobias M. Hohl

**Affiliations:** 1 Louis V. Gerstner Jr. Graduate School of Biomedical Sciences, Sloan Kettering Institute, Memorial Sloan Kettering Cancer Center 5803, New York, New York, USA; 2 Infectious Disease Service, Department of Medicine, Memorial Sloan Kettering Cancer Center 5803, New York, New York, USA; 3 Immuno-Oncology Program, Memorial Sloan Kettering Cancer Center 5803, New York, New York, USA; Instituto Carlos Chagas, Curitiba, Brazil

**Keywords:** *Aspergillus*, neutrophil, innate immunity, antibiotics, ampicillin, microbiota, lung infection, lung defense, fungal infection, molds, reactive oxygen species, monocyte

## Abstract

*Aspergillus fumigatus* is an ubiquitous environmental mold that forms airborne conidia and can cause life-threatening infections in immunocompromised individuals. Invasive aspergillosis occurs in patients with quantitative or qualitative neutrophil defects who often receive systemic antibiotics to prevent or manage bacterial infections. Antibiotic-induced bacterial dysbiosis has been linked to impaired neutrophil bactericidal activity and to intestinal commensal bacteria escape during systemic candidiasis, though it remains unclear whether receipt of antibacterial antibiotics impairs neutrophil-dependent defenses against inhaled mold pathogens in the lung. Herein, we measured the outcome of *A. fumigatus* challenge in C57BL/6J mice that were treated with different antibiotics in the drinking water for 3 weeks prior to experimental infection. We found that ampicillin, but not neomycin or vancomycin treatment, significantly increased murine mortality and lung fungal burden. The heightened susceptibility was associated with impaired fungal killing by lung-infiltrating neutrophils and monocytes, as well as reduced neutrophil production of NADPH oxidase 2 (NOX2)-dependent reactive oxygen species (ROS). These findings demonstrate that systemic antibiotic treatment can compromise pulmonary immunity against *A. fumigatus* and suggest that the host microbiota can enhance neutrophil fungicidal activity by promoting NOX2-mediated ROS production.

**IMPORTANCE:**

*Aspergillus fumigatus* is an environmental mold that causes invasive pulmonary disease in immunocompromised individuals. Owing to limited diagnostic tools, a narrow arsenal of effective treatments, and rising antifungal resistance, the World Health Organization (WHO) has designated *A. fumigatus* as a critical priority fungal pathogen, highlighting the urgent need for further research. Patients with compromised immunity often receive broad-spectrum antibiotics to prevent or treat opportunistic infections, leading to significant disruption of the resident commensal microbiota. This antibiotic-induced dysbiosis has been linked to *Clostridium difficile* colitis and to intestinal overgrowth of vancomycin-resistant *Enterococcus* and *Candida parapsilosis*, preceding bloodstream infection. However, the impact of antibiotic treatment on susceptibility to invasive pulmonary aspergillosis remains undefined. In this study, we found that oral treatment with ampicillin, but not neomycin or vancomycin, significantly increased mortality in mice following *A. fumigatus* infection. Neutrophils from the lungs of ampicillin-treated mice also showed markedly impaired fungal killing. These findings raise the possibility that preserving microbiome integrity during antibiotic treatment could enhance immune protection against invasive aspergillosis in at-risk patient groups.

## OBSERVATION

Phagocytes, such as neutrophils, inflammatory monocytes, and alveolar macrophages, mediate host defense against *Aspergillus fumigatus* (1–3). Phagocyte function during *Streptococcus pneumoniae* and influenza virus pulmonary infections is linked to microbiota composition in mice (4, 5). In addition, antibiotics can increase susceptibility to systemic *Candida albicans* infection (6, 7). These studies suggest that antibiotics shape pulmonary phagocyte responses and influence susceptibility to fungal infection. However, how antibiotic treatment impacts immunity to *A. fumigatus* infection remains unclear.

**Fig 1:**
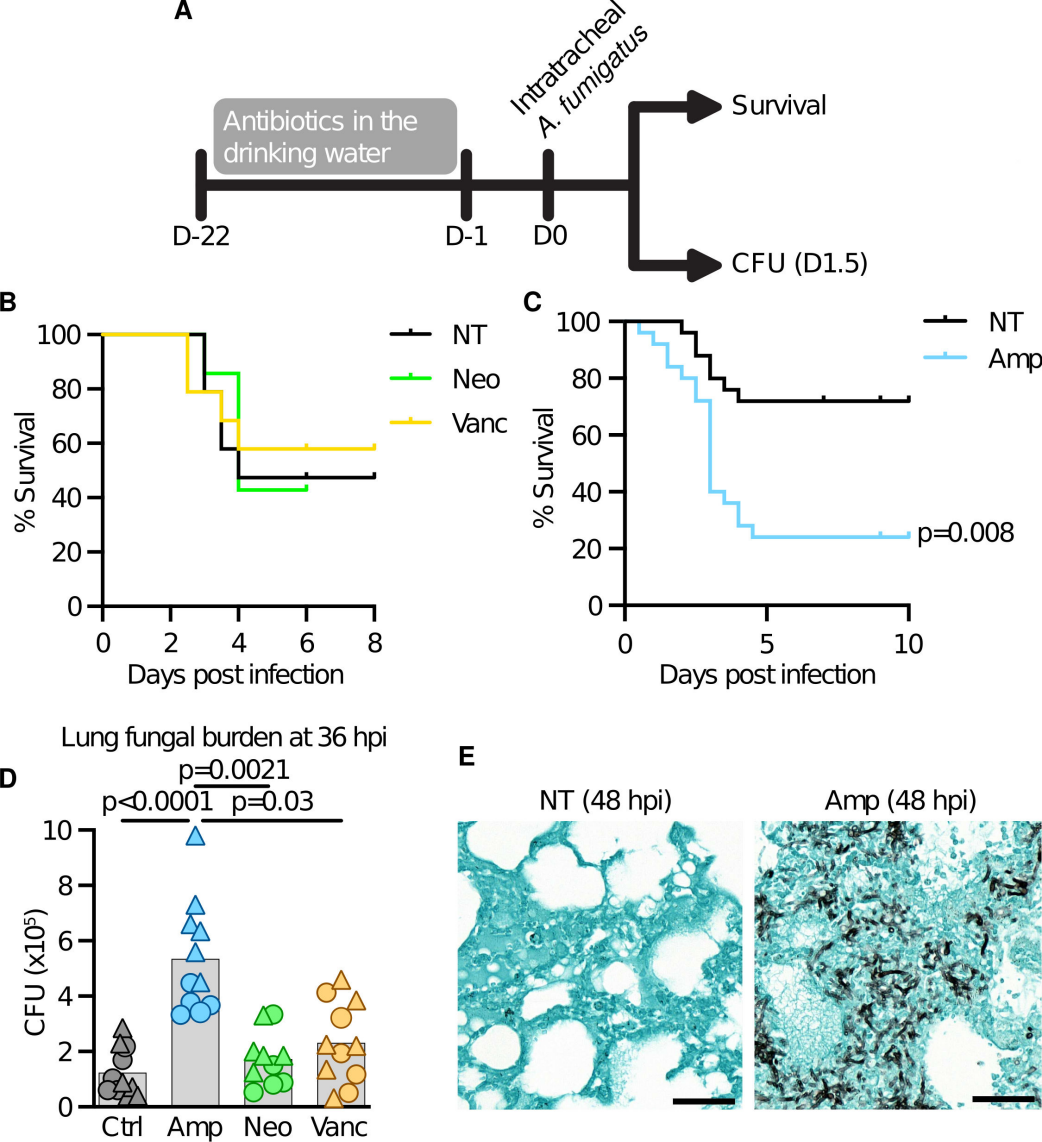
Antibiotic treatment increases susceptibility to *A. fumigatus* infection. (**A**) Schematic of the experimental timeline. (**B and C**) Murine survival following i.t. infection with *A. fumigatus* conidia, comparing not-treated (NT) mice to mice treated with neomycin and vancomycin (**B**) or ampicillin (**C**). Data are pooled from at least two experiments. *n* = 17 in (**B**) and *n* = 25 in (**C**). (**D**) Fungal burden in the lungs at 36 h post-infection, as quantified by CFUs. Data are pooled from two experiments, and point shape indicates experiment of origin. (**E**) Representative histology (Grocott–Gömöri’s methenamine silver stain) of the lungs from control and ampicillin-treated mice 48 h post infection. Statistics: (**B and C**) log-rank (Mantel–Cox) test and (**D**) Kruskal–Wallis test with Dunn’s multiple-comparisons test.

To test if antibiotic treatment increases susceptibility to *A. fumigatus* infection, we treated C57BL/6J (WT) mice with one of three antibiotics in drinking water for 3 weeks. Antibiotics were withdrawn 1 day before intratracheal (i.t.) infection with *A. fumigatus* conidia (Fig. 1A). Control mice received normal drinking water (designated NT). Vancomycin, a glycopeptide primarily targeting gram-positive bacteria, or neomycin, an aminoglycoside primarily targeting gram-negative bacteria, had no effect on survival following pulmonary *A. fumigatus* infection (Fig. 1B). However, treatment with ampicillin, a β-lactam antibiotic that targets gram-positive and -negative and anaerobes that lack β-lactamase activity, resulted in increased mortality following the *A. fumigatus* challenge (Fig. 1C). Additionally, ampicillin-treated but not vancomycin- or neomycin-treated mice had increased lung fungal burden (Fig. 1D), and histopathology analysis revealed greater fungal tissue invasion in ampicillin-treated lungs (Fig. 1E). These findings indicate that ampicillin treatment increases mortality due to increased lung fungal burden.

**Fig 2:**
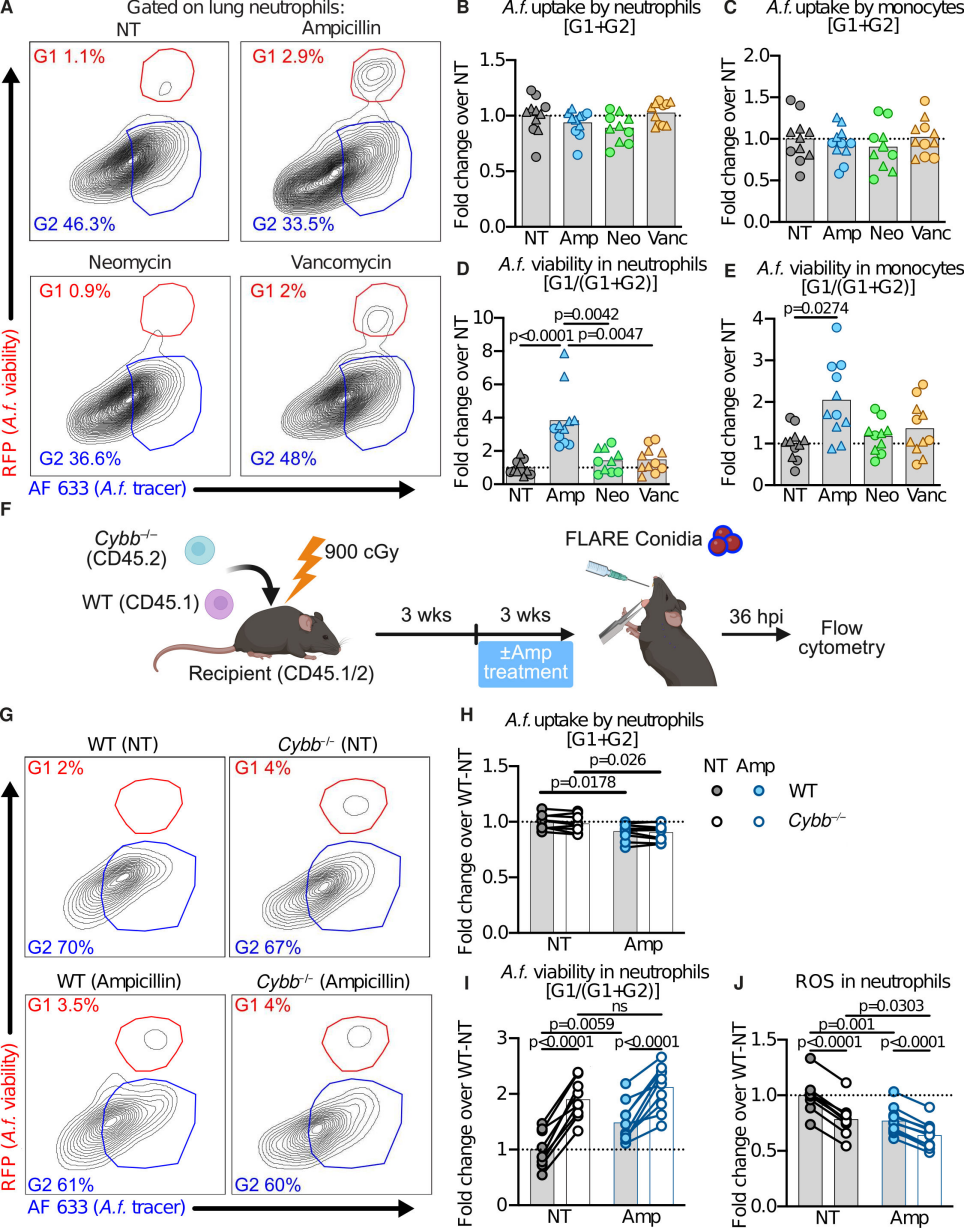
Ampicillin disrupts Nox2–dependent fungal killing by neutrophils. (**A**) Representative flow cytometry plots of lung neutrophils analyzed for RFP and AF633. (**B and C**) Quantification of *A. fumigatus* uptake [G1 + G2] by lung neutrophils (mean ± SD of NT: 55.67 ± 13.94) and lung monocytes (mean ± SD of NT: 36.56 ± 11.15). (**D and E**) Quantification of lung neutrophils (mean ± SD of NT: 2.33 ± 1.26) and lung monocytes (mean ± SD of NT: 1.34 ± 0.89) with live *A. fumigatus* viability [G1 / (G1 + G2)]. (**F**) Schematic of mixed bone marrow chimera generation, followed by ampicillin treatment. Created with BioRender. (**G**) Representative flow cytometry plots of neutrophils analyzed for RFP and AF633 from chimeric mice. (**H**) Quantification of *A. fumigatus* uptake [G1 + G2] by lung neutrophils from chimeric mice (mean ± SD of NT-WT: 68.58 ± 4.65). (**I**) Quantification of *A. fumigatus* viability [G1 / (G1 + G2)] in lung neutrophils from chimeric mice (mean ± SD of NT-WT: 2.86 ± 0.93). (**J**) Quantification of ROS production by lung neutrophils from chimeric mice (mean ± SD of NT-WT: 30,810 ± 4,841). Data in (**B–E**) and (**H–J**) are normalized to the average of the control group. Data in (**B–E**) are pooled from two experiments, and the point shape indicates the experiment of origin. Data in (**H–J**) are from one experiment. Each point in (**B–E**) and (**H–J**) represents a mouse, and the point shape in (**B**) represents the experiment of origin. Statistics: (**B–E**) Kruskal–Wallis test with Dunn’s multiple-comparisons test. (**H–J**) RM two-way ANOVA; matched values are spread across a row and uncorrected Fisher’s LSD with a single pooled variance.

Fungal growth in the lung often reflects defects in neutrophils or inflammatory monocytes (2, 7). To test this, we quantified these cells in the lungs of untreated and ampicillin-treated mice after infection and found no difference at 36 hpi (Fig. S1A and B). We then used fluorescent *Aspergillus* reporter (FLARE) conidia to assess fungal uptake and killing by lung neutrophils and monocytes (8). FLARE conidia encode mRFP and are labeled with Alexa Fluor 633 (AF633). FLARE conidia shift their fluorescence emission from AF633^+^mRFP^+^ to AF633^+^mRFP^–^ when conidia are killed within lung phagocytes. Neutrophil and monocyte conidial uptake (i.e., the frequency of neutrophils and monocytes that contain AF633^+^mRFP^+^ or AF633^+^mRFP^–^ conidia) was similar between antibiotic-treated and untreated mice (Fig. 2A through C). However, lung neutrophils and monocytes from ampicillin-treated mice contained a higher fraction of mRFP^+^ conidia than those from untreated or other antibiotic-treated mice (Fig. 2D and E), indicating a fungal killing defect. Thus, lung fungal clearance in ampicillin-treated mice is impaired due to defective myeloid cell fungal killing, but not defective myeloid cell uptake nor defective myeloid cell recruitment to the lung.

NADPH oxidase 2 (NOX2)-derived reactive oxygen species (ROS) are an essential mechanism used by human and murine neutrophils to kill *A. fumigatus* (9, 10). NOX2 mutations result in greater susceptibility to *A. fumigatus* infection in mice and humans (10, 11), underscoring the importance of this pathway. To test if ampicillin treatment impairs NOX2-dependent killing, we generated mixed bone marrow chimeras by reconstituting irradiated CD45.1^+^CD45.2^+^ mice with a 1:1 mix of CD45.1^+^ WT and CD45.2^+^*Cybb*^–/–^ bone marrow cells. *Cybb* encodes the NOX2 p91 subunit. Mice were either left untreated or received ampicillin during the final 3 weeks of the 6-week reconstitution period and were challenged with 3 × 10^7^ FLARE conidia to assess fungal uptake and killing by WT and *Cybb^–/–^* neutrophils in the same host. Fungal uptake was similar in WT and *Cybb*^–/–^ lung neutrophils, regardless of ampicillin treatment (Fig. 2G and H). In contrast, fungal viability in lung neutrophils differed significantly with both genotype and antibiotic treatment. In untreated mice, a higher frequency of fungus-engaged *Cybb^–/–^* neutrophils contained viable conidia compared to WT neutrophils (Fig. 2G and I), consistent with published results (9). In ampicillin-treated mice, the frequency of WT neutrophils that contained viable *A. fumigatus* conidia increased, mirroring the frequency observed in ampicillin-treated non-chimeric mice (Fig. 2D and I). In contrast, *Cybb^–/–^* neutrophils did not show an increase in fungal viability with ampicillin treatment. Ampicillin, thus, impairs neutrophil fungal killing by disrupting NOX2-dependent fungal killing. Supporting this finding, ampicillin treatment reduced ROS production in WT lung neutrophils and, to a lesser extent, in *Cybb^–/–^* neutrophils (Fig. 2J).

Because ampicillin has been reported to directly scavenge ROS produced by neutrophils (12), we tested its direct effects on antifungal activity using FLARE conidia. Incubating bone marrow cells with increasing concentrations of ampicillin spanning peak serum levels in rodents (13) had no effect on neutrophil viability, neutrophil fungal uptake, or neutrophil fungal killing (Fig. S2). Treatment with the vacuolar ATPase inhibitor, bafilomycin A1, resulted in increased fungal viability in neutrophils, consistent with prior studies in macrophages (1) and confirming that the assay could detect impaired fungal killing. Thus, ampicillin does not directly impair neutrophil antifungal activity.

Our findings add to the literature demonstrating that antibiotic-induced microbiome dysbiosis impairs innate immune defense against pathogen challenge. Germ-free or antibiotic-treated mice exhibit reduced neutrophil production and increased susceptibility to *Escherichia coli* and *Listeria monocytogenes* infection (14, 15). However, we observed no change in neutrophil accumulation in the lung following *A. fumigatus* infection. Similarly, prior work found that neutrophils from antibiotic-treated mice retain normal phagocytic capacity, which we confirm in the context of *A. fumigatus* (14). Instead, we identify a selective defect in fungal killing, consistent with a report that antibiotic treatment can impair neutrophil antimicrobial function against *S. pneumoniae* (16). Here, we identify reduced NOX2-dependent ROS production as a mechanism by which antibiotic treatment impairs neutrophil antimicrobial activity.

Vancomycin treatment inhibits IL-17 production during *A. fumigatus* infection (17). However, this effect was not associated with impaired fungal clearance or mortality, consistent with our findings (Fig. 1B and D). IL-17 levels in ampicillin-treated mice were similar to untreated mice (Fig. S3A). During *S. pneumoniae* lung infection, antibiotic treatment reduced GM-CSF production (4). Notably, GM-CSF is essential for immunity to *A. fumigatus* (18), but GM-CSF levels were elevated, not diminished, in ampicillin-treated mice after *A. fumigatus* infection (Fig. S3B). This trend extended to other cytokines examined (Fig. S3C through M), which were either comparable to or higher than in untreated controls. These data indicate that increased susceptibility arises from impaired phagocyte effector function rather than altered cytokine signaling.

Susceptibility to *A. fumigatus* infection was specific to ampicillin and not observed with neomycin or vancomycin, suggesting that depletion of anaerobic bacteria preferentially targeted by ampicillin (19) contributes to impaired lung neutrophil function. Future studies should focus on linking perturbations of specific commensal bacteria taxa and dependent metabolites, including colonization of candidate taxa in germ-free mice, to regulation of NOX2 activity during *A. fumigatus* infection.

A limitation of this study is the 24–30-h half-life of ampicillin in aqueous solution (20). Thus, weekly replacement of ampicillin drinking water may not have achieved maximal depletion of ampicillin-sensitive bacterial taxa. Nevertheless, we observed a clear impact on *A. fumigatus* susceptibility and neutrophil function. Increasing the dosing frequency and assessing microbiota depletion by 16S qPCR would be desirable in future studies that involve antibiotic administration.

This study establishes a link between antibiotic treatment and increased susceptibility to *A. fumigatus* infection, providing mechanistic insight into how antibiotic treatment impairs pulmonary antifungal immunity. These findings underscore the potential clinical relevance of antibiotic stewardship in immunocompromised individuals, who are at elevated risk for invasive aspergillosis, and suggest that selecting antibiotics that preserve microbiome integrity may mitigate infection-related morbidity and mortality in these settings.

## Data Availability

Requests for further information, resources, or reagents should be directed to the corresponding author.

## SUPPLEMENTAL MATERIAL

Supplemental Material mbio.03982-25-s0001.docx Supplemental materials and methods and Fig. S1–S3.

ASM does not own the copyrights to Supplemental Material that may be linked to, or accessed through, an article. The authors have granted ASM a non-exclusive, world-wide license to publish the Supplemental Material files. Please contact the corresponding author directly for reuse.
