# Supplementary material for: Antibiotic-induced microbiota disruption impairs neutrophil-mediated immunity to respiratory Aspergillus fumigatus infection in mice
Source: mBio. 2026 Mar 11;17(4):e03982-25. doi: 10.1128/mbio.03982-25 (PMC13059706; doi:10.1128/mbio.03982-25)
Supplement: Supplemental Material — s and methods and Fig. S1–S3. [file mbio.03982-25-s0001.docx]

**SUPPLEMENTAL MATERIALS**

**SUPPLEMENTAL METHODS**

**Mice**

C57BL/6J wild-type (cat#: 000664) and *Cybb*^–/–^ mice (cat#: 002365) were obtained from The Jackson Laboratory (Bar Harbor, ME, USA). CD45.1^+^ mice (stock# 564) were purchased from Charles River Laboratories. CD45.1^+^CD45.2^+^ were generated by crossing CD45.1^+^ mice to C57BL/6J mice. Mice used in this study were 8-12 weeks old. Within experiments, mice were age- and sex-matched. Experiments were performed with both male and female mice. Mice were bred and housed in the Research Animal Resource Center at MSKCC in individual ventilated cages under specific pathogen–free conditions. Animal experiments were conducted with approval of the MSKCC Institutional Animal Care and Use Committee under the protocol 13-07-008.

**Antibiotic treatment**

Antibiotics were administered to mice via drinking water for 3 weeks at the following concentrations: 1 g/L ampicillin, 1 g/L neomycin, or 0.5 g/L vancomycin as previously described (6). All antibiotics were purchased from the MSKCC pharmacy. Solutions were replaced weekly. The day prior to infection, mice were returned to normal drinking water.

**Mouse infections**

*A. fumigatus* strain CEA10 (provided by R. Cramer, Dartmouth College) was used for all experiments. For experiments to analyze fungal uptake and killing using FLARE conidia, the CEA10–monomeric RFP strain was used. *A. fumigatus* conidia were grown on glucose minimal medium slants for 4–7 days at 37 °C before harvesting in phosphate-buffered saline (PBS) + 0.025% Tween 20 for experimental use. For FLARE experiments, 7.5 × 10^8^ conidia were incubated in EZ-Link Sulfo-NHS-LC-Biotin (10 μg/ml; Thermo Fisher Scientific) in 1 ml of 50 mM NaHCO3 buffer (pH 8.3) for 1–2 hours at room temperature, washed with 1 ml of Tris-HCl (pH 8) buffer, incubated with streptavidin (20 μg/ml) and Alexa Fluor 633 conjugate (Molecular Probes) in PBS for 45 minutes at room temperature, and resuspended in PBS + 0.025% Tween 20. Mice were anesthetized by isoflurane inhalation, and 3–6 × 10^7^ *A. fumigatus* conidia were instilled via the intratracheal route in 50 μl of PBS + 0.025% Tween 20.

**Quantification of lung fungal burden**

To measure lung fungal burden, lungs were dissected and homogenized with a PowerGen 125 homogenizer (Thermo Fisher Scientific) for 10–15 seconds in 2 ml of PBS. A total of 10 μl was removed and diluted for plating onto Sabourand dextrose agar. Plates were incubated for 48 hours at 37 °C, and CFU were enumerated.

**Histology**

Mouse lungs were collected into histology cassettes, stored in 4% paraformaldehyde for 16 hours, and then placed in ethanol. Samples were embedded in paraffin, sectioned, and stained with Grocott–Gömöri's methenamine silver stain (GMS) to identify fungal organisms. Slides were scanned using a Pannoramic Digital Slide Scanner (3DHISTECH) using a 20×/0.8 numerical aperture objective.

**Flow cytometry**

For analysis of immune cells, single-cell suspensions of mouse lungs were generated by putting lungs in gentleMACS C tubes and mechanically homogenizing in 5 ml of PBS using a gentle MACS Octo Dissociator (Miltenyi Biotec) in the absence of enzymes and then filtered through 100-μm filters. Red blood cells were lysed using red blood cell lysis buffer (Tonbo Biosciences), cells were blocked with anti-CD16/CD32, stained with fluorophore-conjugated antibodies, and analyzed on a Beckman Coulter Cytoflex LX. Single-color controls for compensation were generated using lung cells or OneComp eBeads Compensation Beads. Experiments were analyzed with FlowJo version 10.8.1. Dead cells were excluded with 4′,6-diamidino-2-phenylindole (DAPI) or eBioscience Fixable Viability Dye eFluor 506 (Thermo Fisher Scientific). The antibodies used are the following: anti-Ly6C (clone AL-21), anti-Ly6G (clone 1A8), anti-CD11b (clone M1/70), anti-CD11c (clone HL3), anti-CD45 (clone 30-F11), anti-CD45.1 (clone 104), anti-CD45.2 (clone A20), anti-I-A/I-E (clone M5/114.15.2), and anti-Siglec-F (clone E50-2440) all from Biolegend or BD Biosciences. Inflammatory monocytes were identified as CD45^+^ CD11b^+^ CD11c^–^ Siglec-F^–^ Ly6G^–^ Ly6C^hi^ MHC-II^–^ cells and neutrophils were identified as CD45^+^ CD11b^+^ Siglec-F^–^ Ly6G^+^. For experiments with mixed bone marrow chimeras, WT cells and *Cybb*^–/–^ cells were distinguished by CD45.1 and CD45.2 expression, respectively.

Conidial uptake and viability in lung phagocytes were assessed as previously described (8). After gating on lung monocytes or lung neutrophils, uptake was defined as the sum of RFP⁺AF633⁺ and RFP⁻AF633⁺ cells within each population. Conidial viability within a given phagocyte subset was calculated as the proportion of cells containing live conidia (RFP⁺AF633⁺) relative to all conidia-engaged cells (RFP⁺AF633⁺ and RFP⁻AF633⁺).

**Bone marrow chimeras**

To generate mixed bone marrow chimeras, CD45.1^+^CD45.2^+^ recipient mice were irradiated (900 centigray) and reconstituted with a 1:1 mixture CD45.1^+^ wild-type and CD45.2^+^ *Cybb^–/–^* bone marrow totaling 4 × 10^6^ donor bone marrow cells. After bone marrow transplantation, recipient mice were rested for 6 weeks prior to experimental use. Control mice received no antibiotics during this period, while ampicillin-treated mice were given antibiotics during the final 3 weeks, as described above.

**ROS Assay**

Reactive oxygen species (ROS) production in lung neutrophils was measured using Dihydrorhodamine 123 (DHR; Invitrogen). Lung cells were incubated with 100 µM DHR, anti-Ly6G, and anti-CD11b antibodies in HBSS for 20 minutes at 37 °C. Dead cells were excluded with DAPI, and samples were analyzed on a Beckman Coulter Cytoflex LX.

**Ampicillin dose-response FLARE assay**

Bone-marrow cells were harvested from the femurs and tibias of C57BL/6J mice, subjected to ACK lysis, and 1 × 10^6^ cells were seeded in a 24-well plate in complete RPMI-1640 (10 % heat-inactivated FBS, 2 mM L-gutamine, 1 mM sodium pyruvate, 10 mM HEPES, and non-essential amino acids. Pen/Strep was not added to the media for this experiment. Cells were pre-treated for 30 min at 37 °C with ampicillin (0, 1, 3, 10, or 30 µg/mL). Bafilomycin A1 (50 nM) served as an impaired-killing control. FLARE conidia were added at a MOI of 1. To prevent fungal germination, voriconazole (500 ng/mL) was added to the media. Following overnight culture, cells were labelled with anti-Ly6G, and anti-CD11b antibodies. Dead cells were excluded using DAPI. Samples were analyzed on a Beckman Coulter Cytoflex LX.

**Quantification of lung cytokines**

Lungs were harvested and homogenized in 2 mL of PBS using a PowerGen 125 homogenizer (Thermo Fisher Scientific) for 10–15 seconds. Homogenates were centrifuged, filtered through a 40 µm cell strainer, and stored at –80 °C until analysis. Cytokine levels were measured using the LEGENDplex™ Mouse Inflammation Panel (13-plex) (BioLegend) according to the manufacturer’s instructions.

**SUPPLEMENTAL FIGURES**

| 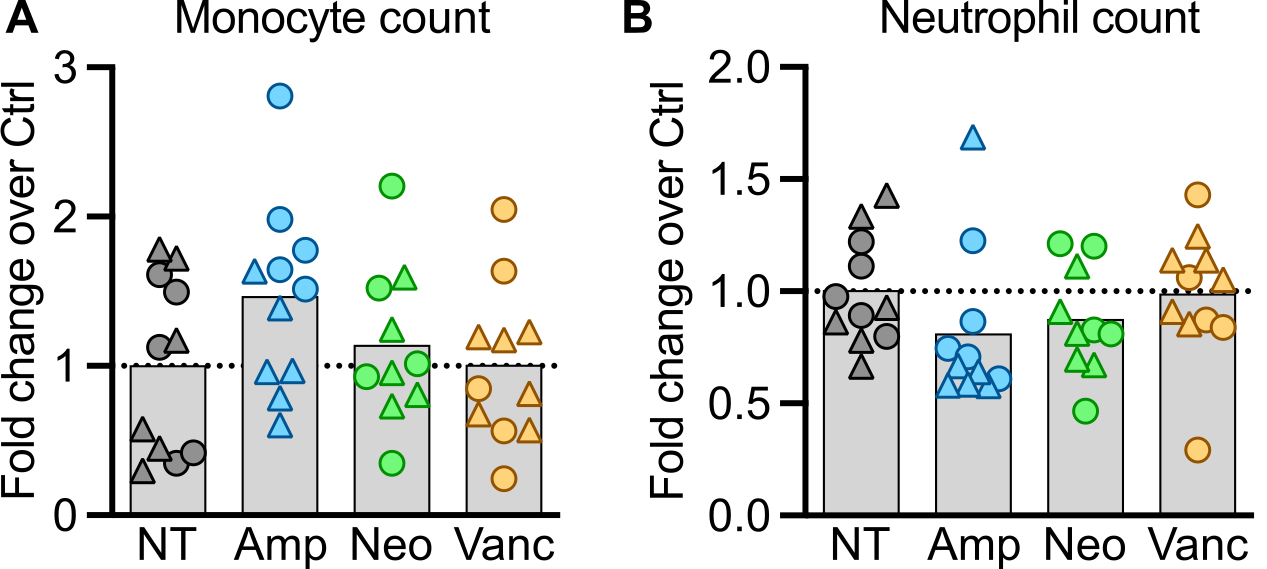 |
| --- |
| **Figure S1: Quantification of lung phagocyte abundance and fungal uptake following** *A. fumigatus* **infection.**  (A) Quantification of monocytes (Mean ± SD of NT: 104201 ± 66677) and (B) neutrophils (Mean ± SD of NT: 1632403 ± 444446) from the lungs of control or antibiotic-treated mice 36 hours after *A. fumigatus* infection.  Statistics: Kruskal–Wallis test with Dunn’s multiple comparisons test. |
| 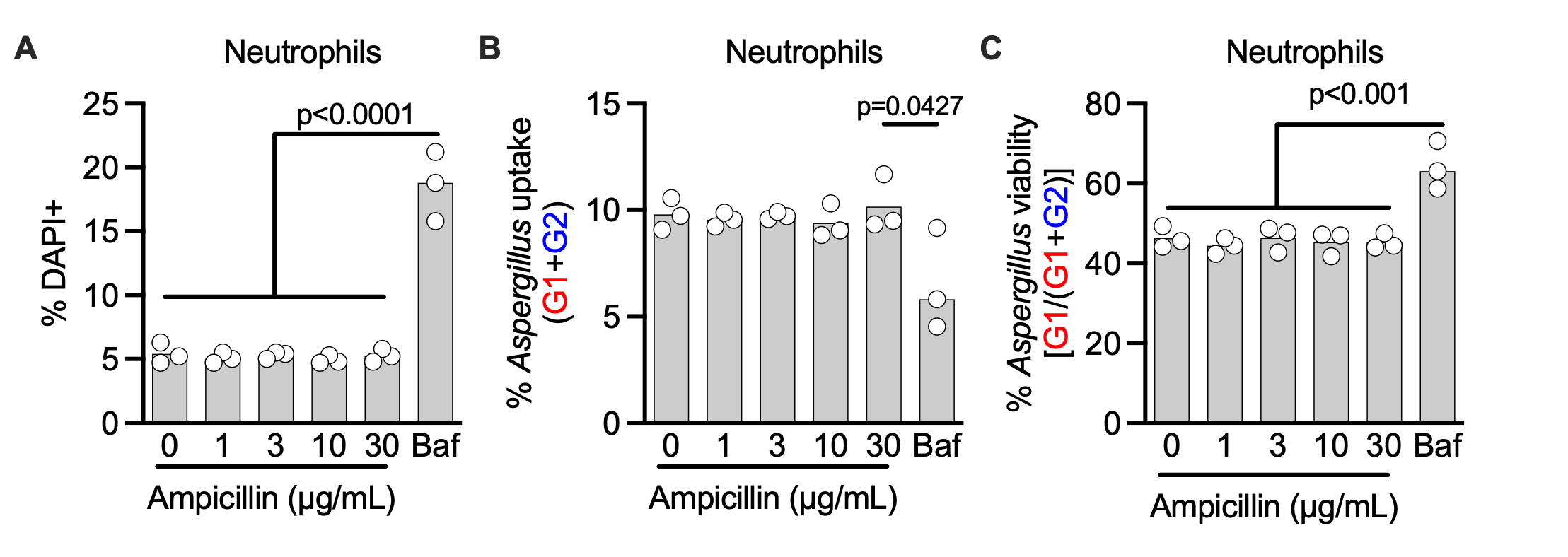 |
| **Figure S2: Ampicillin does not inhibit fungal killing by neutrophils in vitro.**  (A) Quantification of bone marrow neutrophil viability after overnight culture with FLARE conidia.  (B) Quantification of *A. fumigatus* uptake by DAPI^–^ bone marrow neutrophils.  (C) Quantification of *A. fumigatus* viability by DAPI^–^ bone marrow neutrophils.  Data are representative of two independent experiments. Each point indicates a mouse. Statistics: Ordinary one-way ANOVA and Dunnett’s multiple comparisons test, with a single pooled variance. |
| 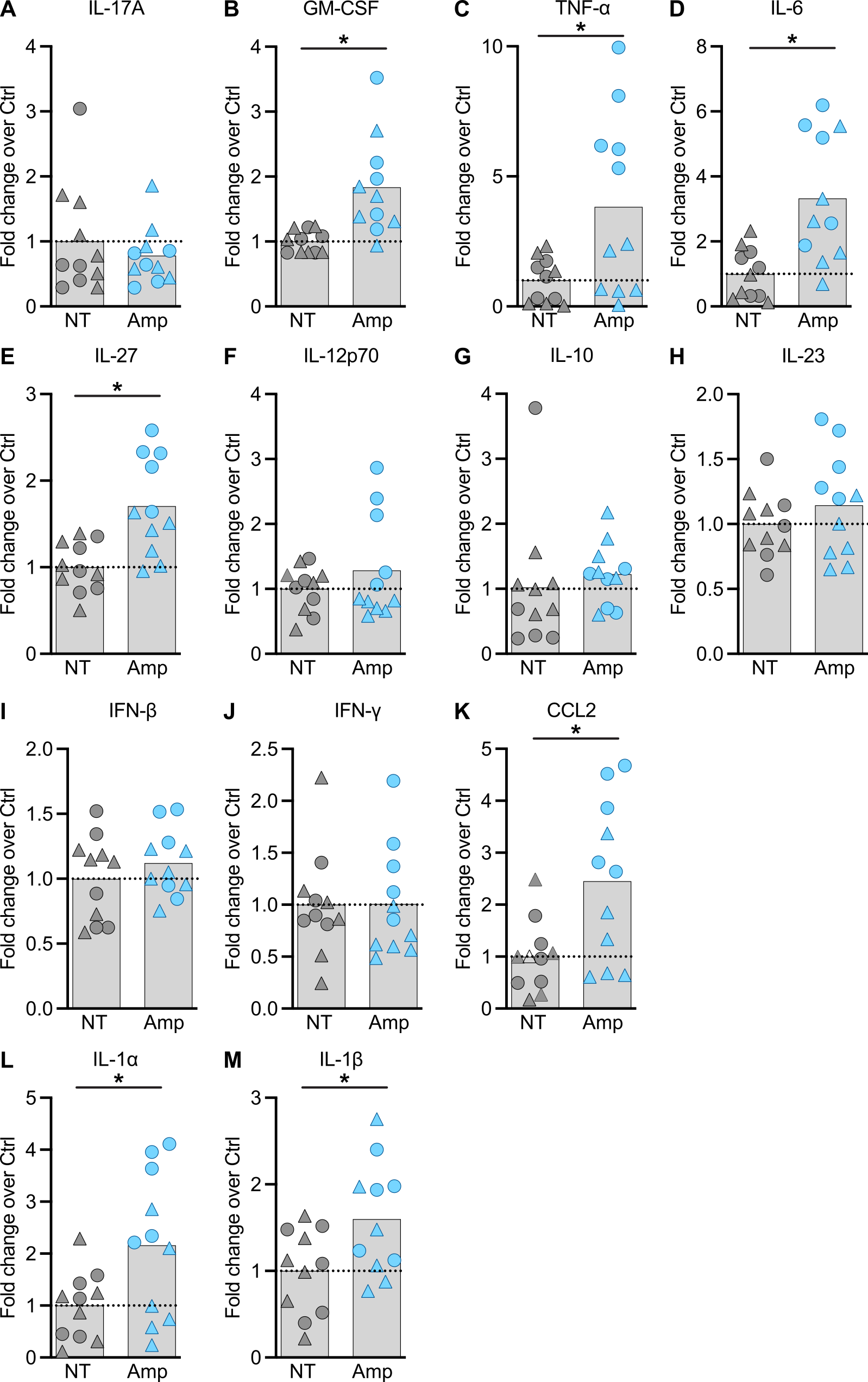 |
| **Figure S3: Lung cytokine levels in ampicillin-treated or control mice 36 hpi with *A. fumigatus.***  Data in (A-F) are normalized to the average of the control samples. Data are pooled from two experiments, and the point shape indicates the experiment of origin.  Statistics: Unpaired t tests with Welch correction on each row, with multiple comparisons corrected using the Benjamini, Krieger, and Yekutieli procedure (FDR = 5%). Asterisks indicate differences that remained statistically significant after correction for multiple comparisons. |
